# Experiment in Liquefying Gases

**Published:** 1874-12

**Authors:** 


### EXPERIMENT IN LIQUEFYING GASES.

A piece of fresh wood charcoal is placed in a strong U tube like that first used by Faraday in his researches upon the condensation of gases. This is to be saturated with dry chlorine, ammonia, or other gas which is desired to experiment upon. The two ends of the tube are then sealed before the blowpipe. The longer end of the tube, which contains the charcoal, is now to be heated in a boiling salt water bath and the shorter one plunged into a freezing mixture. The result will be the speedy evolution of the absorbed gas from the charcoal, and its condensotion by its own pressure in the colder portions of the tube. It is thus possible to prepare very quickly a quantity of liquefied gas for a lecture experiment. On taking ont the tube from the salt bath, the liquid chlorine (or other substance) begins to boil spontaneously, and is again absorbed by the charcoal, while the short end of the tube becomes covered with frost, from the rapid abstraction of heat from it, by the passage of the liquid into the gaseous state.

In this way it is possible to prepare, in the liquid state, chlorine, ammonia, sulphurous acid, sulphuretted hydrogen, hydrobromic acid, chloride of ethyl, cyanogen, and other gases. —*Boston Journal of Chemistry.*

